# Land-Use Change and Emerging Infectious Disease on an Island Continent

**DOI:** 10.3390/ijerph10072699

**Published:** 2013-06-28

**Authors:** Rosemary A. McFarlane, Adrian C. Sleigh, Anthony J. McMichael

**Affiliations:** National Centre for Epidemiology and Population Health, Australian National University, Canberra ACT 0200, Australia; E-Mails: adrian.sleigh@anu.edu.au (A.C.S.); tony.mcmichael@anu.edu.au (A.J.M.)

**Keywords:** emerging infectious disease, zoonoses, wildlife, livestock, vector, environment, land-use, Australia

## Abstract

A more rigorous and nuanced understanding of land-use change (LUC) as a driver of emerging infectious disease (EID) is required. Here we examine post hunter-gatherer LUC as a driver of infectious disease in one biogeographical region with a compressed and documented history—continental Australia. We do this by examining land-use and native vegetation change (LUCC) associations with infectious disease emergence identified through a systematic (1973–2010) and historical (1788–1973) review of infectious disease literature of humans and animals. We find that 22% (20) of the systematically reviewed EIDs are associated with LUCC, most frequently where natural landscapes have been removed or replaced with agriculture, plantations, livestock or urban development. Historical clustering of vector-borne, zoonotic and environmental disease emergence also follows major periods of extensive land clearing. These advanced stages of LUCC are accompanied by changes in the distribution and density of hosts and vectors, at varying scales and chronology. This review of infectious disease emergence in one continent provides valuable insight into the association between accelerated global LUC and concurrent accelerated infectious disease emergence.

## 1. Introduction

Land-use change (LUC) is reported to be a major driver of emerging infectious diseases (e.g., [1,2,3]). Indeed, LUC, food production and agricultural change are reported to collectively account for almost half of all global zoonotic EIDs [3]. However there is some lack of consistency in the use this term, elsewhere defined simply as the management of land to meet human needs [4]. Such management includes agriculture, forestry, mining and all forms of urban and industrial use. EIDs have been recognised and described since the 1970s and refer to novel or known infections undergoing changes in pathogenicity or geographic range [1]. Accelerated, global LUC and natural ecosystem disruption is a concurrent feature of the second half of last century [5]. However, a finer grained definition of LUC as a driver of EIDs is required to improve our understanding of these relationships and, ultimately, our capacity to make land-use policy that optimises public health outcomes.

Ecological change arising from LUC is of particular interest as the majority of EIDs are zoonoses, the majority of these from wild animals [6,7]. Zoonotic disease emergence may be influenced by changes in the density or abundance of hosts, vectors and pathogens; changes in the species composition of communities; changes in life cycles and exposure pathways and by selection pressure for increased pathogen virulence [8]. These changes in host and pathogen are influenced by landscape change, most often anthropogenic (*i.e.*, LUC), which may occur at multiple spatial and temporal scales and patterns [9], and vary in response to different underlying abiotic and biotic elements. Land-use and vegetation cover change (*i.e.*, LUCC) is one measure of ecological change that can be quantitatively described using remotely sensed, spatially and temporally explicit data on land cover. However data on LUCC at the location of emergence may not be of adequate scale to explain emergence [10]. For many diseases, the scale at which ecological change affects emergence is not known.

Associations between ecological change and infectious disease cycles are complex and a challenge for epidemiology [11,12,13,14,15,16,17]. The complex associations between LUC and EIDs have mostly been explored within specific disease studies. These include, for example: avian influenza, domestic ducks and rice agriculture in Thailand [18]; landscape fragmentation and hantavirus in Canada [19]; haemorrhagic fever and renal syndrome and changes of land-use in the Three Gorges region, China [20]; reforestation and Lyme disease in the USA [21]; development roads and diarrhoeal diseases in rural Ecuador [22]. Some reviews have examined EIDs associated with specific LUC types, e.g., deforestation [23] or large dams [24]. Others have looked at the effect of LUC on specific pathogen groups [25,26].

Here we examine the contribution of LUC to infectious disease emergence in a single continent. We do this with reference to Australia, an island nation with just 225 years of post hunter-gatherer LUCC brought about by European penal colonisation in 1788. In Australia, this post hunter-gatherer period is condensed into a much shorter and better documented period than most of the rest of the world. In the intense period of LUCC since colonisation, Australia has lost approximately 50% of its forest and woodland cover and most of the rest is degraded [27]. It has gained the highest extinction rate in the developed world for its mammals, predominantly highly endemic marsupials [28]. Agricultural and pastoral enterprises account for 61% of land-use and there is an extensive mining sector. The majority (87%) of the population of 22 million is concentrated in urban centres [29]. These occur predominantly in the high rainfall, biodiverse, coastal regions. Novel zoonotic vector-borne diseases and diseases of wildlife origin feature prominently in previous reviews of EIDs in Australia [30,31].

In this study, we examine all EIDs of humans and animals for Australia identified by a systematic review of literature spanning the years 1973–2010, thereby creating a unique overview. We also examine the history of infectious disease emergence from European settlement (1788) to the current era of EID concern. Evidence of LUCC-EID effects is categorised using a system of LUCC classification. Our investigations cover a short but complete history of rapid modern LUCC within one unique biogeographic and political region. Therefore we are able to present a short but complete analysis of the influence of this LUCC on EIDs in one region that that can be used to consider LUCC as a driver of EIDs in other regions or nations with less condensed or isolated LUCC history.

## 2. Methods

### 2.1. Definitions and Boundaries

This study is limited to EIDs of humans and terrestrial vertebrate domestic and wild animals in mainland Australia and Tasmania. To manage data we grouped EIDs by three classification variables as follows: type of host infected (human, domestic animal or wildlife); source of the pathogen (environment, wildlife, domestic animal or human); vector-borne (yes or no).

LUCC definition and categories used for analysis in this study were: RESIDUAL native vegetation (e.g., conservation reserves and other natural areas); MODIFIED native vegetation (occasional, low to medium grazing and selective logging); TRANSFORMED (intensive grazing and tree removal including degraded and abandoned land); REPLACED (intensive animal husbandry, horticulture and plantations); REMOVED (settlements and infrastructure). These sequential LUCC categories follow the Vegetation Assets, States and Transitions (VAST) system and its assigned Australian Land Use Management Classifications (Version 7, May 2010) [32]. This transition sequence is also discussed elsewhere (e.g., [33,34]) and provides some information on hydrological change (e.g., removal of vegetation by dam building, replacement of vegetation for wetland modification) although does not address this process specifically. In addition we note the scale of LUCC that effects disease emergence (immediate, local, regional, national) and scale of infection risk (individual, population or global).

### 2.2. Sources of Data on EIDs, Study Procedures and Exclusions

EIDs included in this review are derived from a systematic literature review using databases Scopus (includes 100% of Medline), CAB and Web of Science from 1973–2010. Search terms were: emerging infectious disease(s) and/or emerging communicable disease(s) by region Australasia and country Australia.

Grey literature on EIDs was also consulted from the following organisations: Australian Wildlife Health Network; Office International des Epizooties (Australia); Department of Agriculture Fisheries and Forestry (Australia); ProMED-mail (October 1994 to December 2009). This reduced publication bias by ensuring EIDs of animals were recorded.

**Figure 1:**
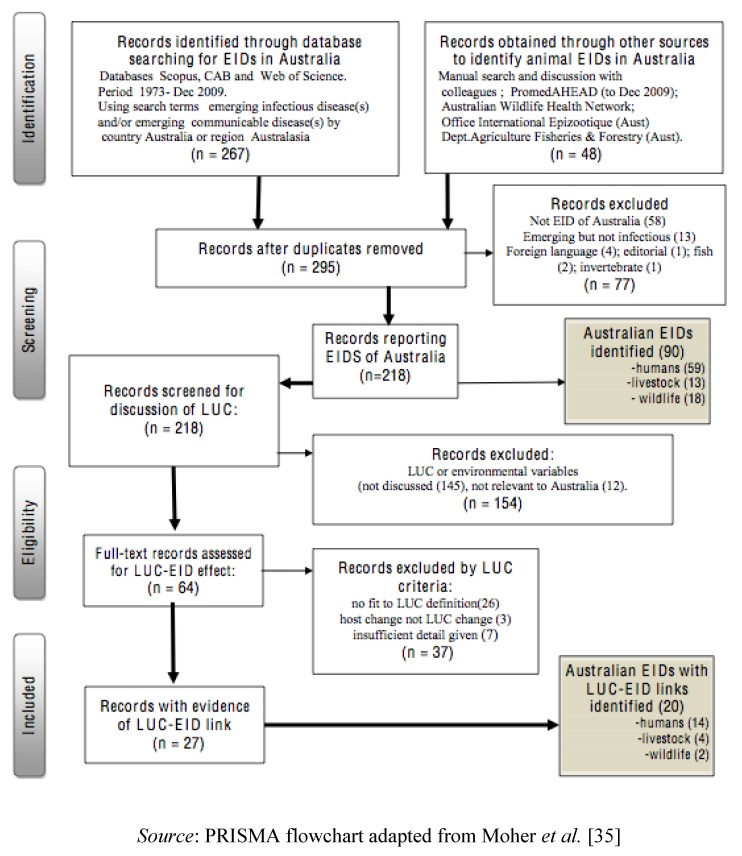
Data flow diagram showing systematic review process: does land-use and cover change influence emerging infectious disease in Australia.

The PRISMA process for Systematic Reviews was followed to identify and process information about EIDs associated with LUCC, as described in a data flow diagram (Figure 1) [35]. In the first stage we excluded papers reporting diseases that were not EIDs or not Australian EIDs. To make the task manageable we excluded infections of fish, invertebrates and plants. Editorials were excluded because they are often speculative and based on selected sources. Papers in foreign languages were excluded because we lacked the resources to appraise thoroughly. In the second stage we excluded EID papers with no reference to land-use or ecological change, and where potential LUCC-linked pathogens of environmental origin emerged in human HIV/AIDs patients primarily as a result of increased host susceptibility.

By full text screening of remaining records we note when there is evidence of an association between LUCC and EID. This assessment was done by one of us (RM) who used the following three criteria to determine if there was evidence of an LUCC-EID association: (1) suggested by author; (2) biologically and ecologically plausible in the context described in the record; (3) temporarily sequenced, with LUCC preceding the EID. Using this method we could apply fundamental criteria of causality to assess associations. When a paper fulfilled all three criteria, the LUCC-EID link was accepted. For each EID these associations were classified according to the single most dominant category of LUCC. This enabled analysis of LUCC categories as drivers of EIDs. As well the scale of LUCC that affects disease emergence and the scale of infection risk were noted (see Definitions above).

As a separate process, run in parallel to the formal Systematic Review, historical information was also sought on LUCC and infections which emerged or re-emerged in the period 1788–1973. Diverse literature (No. of articles in brackets) was hand-searched and comprised the following: medical histories of Australia including paleopathology, colonial and indigenous health (10); historical reviews of specific diseases or diseases groups (7); veterinary histories (2); theses (2); agricultural histories (3); agricultural biography (1); land use and vegetation change in government reports (5), books (1) and web sites (2). These sources are listed in Supplementary Material Appendix 1. As well, discussion with expert colleagues was used to cross check information on infectious diseases that emerged in Australia following European colonisation, noting those that emerged from indigenous pathogens, and those associated with LUCC.

## 3. Results and Discussion

### 3.1. Results

#### 3.1.1. Results of Systematic Literature Review

The review identified 267 papers and 48 grey records bearing on EIDs. After exclusions a total of 218 records remained with an initial total of 90 EIDs (59 diseases of humans (30 zoonotic) 12 diseases of domestic animals and 18 diseases of terrestrial wildlife) that met the criteria for emerging or re-emerging diseases in Australia since 1973. A list of these diseases and key references are available as Online Supplementary Information, Table 1. They include both newly identified and re-emerging diseases.

After screening for mention of land-use or ecological change 64 papers remained. Further selection of this material (see Methods) for information linking LUCC and EIDs (Figure 1) produced 27 papers concerning 20 EIDs caused by 18 pathogens (Table 1). Of the 20 EIDs there were 14 EIDs of humans, four EIDs of domestic animals and two EIDs of wildlife. The majority of the 18 EID pathogens were of environmental (n = 4) or wildlife (vector-borne (n = 5), no vector (n = 3)) origin. A total of six pathogens were vector-borne diseases. Some pathogens cause diseases of both humans and animals (n = 8), two of which, Hendra virus and Menangle virus are of considerable concern as both human and animal diseases. As well we noted a substantial number of the 14 human EIDs were zoonotic (n = 9). 

With reference to the full set of 90 EIDs identified, 22% (20/90) had evidence of LUCC-EID association. Of all the human EIDs, 24% (14/59) were associated with LUCC; the corresponding LUCC proportions for EIDs of domestic animals and wildlife were 33% (4/12) and 11% (2/18), respectively. In addition, the proportion of the 30 zoonotic EIDs with LUCC-EID associations was 30% (9/30).

Statistical evidence linking LUCC to disease emergence is reported for melioidosis in the Northern Territory [36]. Other environmental investigations of LUCC-EID have also produced positive results (e.g., artificial wetlands and parks in coastal developments in Victoria have been shown to have high levels of *Mycobacterium ulcerans* [37,38]; land degradation (dry land salinity) has been shown to increase the vector-borne risk for Ross River virus in south west Western Australia [39]).

**Figure 2:**
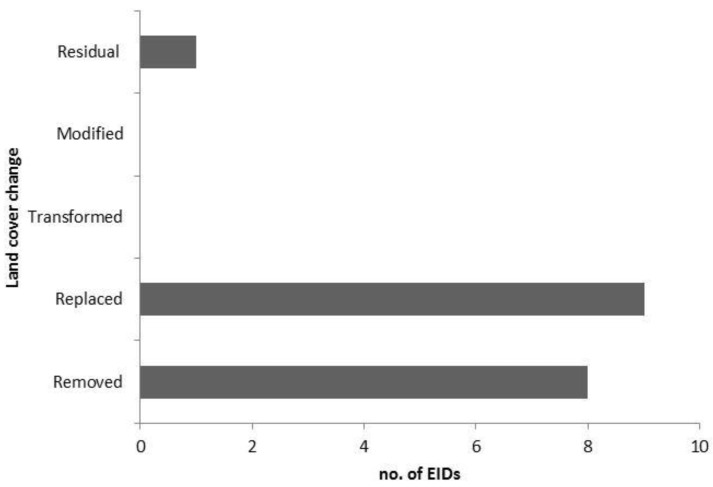
Land cover change: emerging infectious disease associations in Australia based on a systematic review of literature 1973–2010.

The LUCC processes described for the 18 EID pathogens are shown in Table 1. The breakdown of primary LUCCs is displayed in Figure 2. The LUCC-EID associations reported referred to Queensland (n = 11), New South Wales (n = 7) Victoria (n = 6) Western Australia (n = 7), Northern Territory (n = 4) South Australia (n = 2) Tasmania (n = 5). The LUCC-EID associations were discussed as acting at a regional (n = 10) or local (n = 8) scale affecting population (n = 10) or individual (n = 8) risk of infection.

#### 3.1.2. Historical Perspective

**Figure 3:**
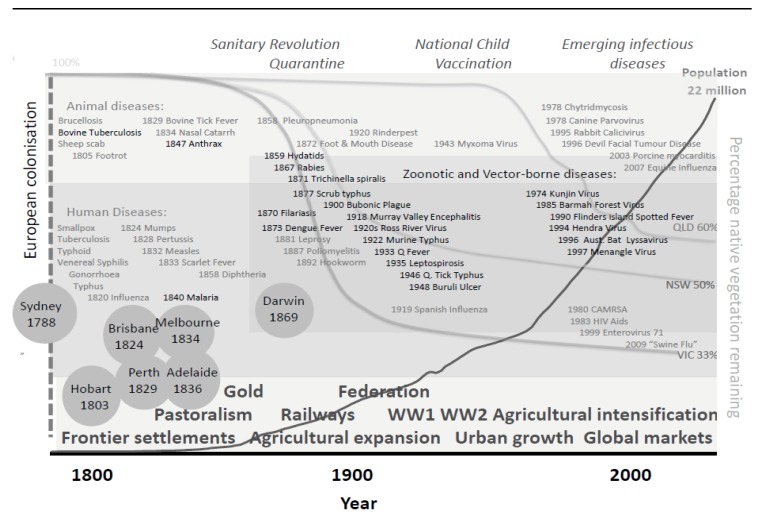
The history of infectious diseases emergence in Australia 1788–2009 with key development and public health events. Data compiled from historical literature and systematically reviewed literature (see Online Supplementary Information, Appendix 1 and Table 1 for full list). Australian population [40] and native vegetation clearance in New South Wales (NSW), Victoria (Vic) and Queensland (Qld) [27] also shown.

The historical perspective provides a political, socio-economic and ecological background to infectious diseases emergence in Australia that contextualises the more recent history of EIDs and LUCC, above. Many diseases now regarded as re-emerging in Australia were first identified following major agricultural expansion in eastern Australia. The chronology of disease emergence and native vegetation clearing, against key development and public health events, can be considered concurrently (Figure 3), and as summarised below.

**Table 1:** Emerging Infectious Diseases (EID) of Australian humans and animals with associations with land use change (LUCC) (n = 20) based on a systematic review of literature 1973–December 2009.

Pathogen	Host	Location	LUCC associated with emergence described in literature
**Origin: environment **			
Meliodosis *Burkholderia pseudomallei* *	H D W	NT (endemic) other foci e.g., s.w. WA	**(4):** Increased soil disturbance through gardening, farming soil cultivation, livestock, irrigation [36,41]
Buruli Ulcer * Mycobacterium ulcerans	H D W	Coastal Vic & Q	**(5):** Nutrient enrichment in coastal developments (e.g., golf courses, storm water drainage) [37,38]
Cryptococcosis *Cryptococcus gattii* *	H D W	Regional mainland Australia	**(4):** Plantations, residence adjacent to plantations or naturally occurring host trees (River Red Gums *Eucalyptus camaldulensis*) [42]
*Photorabdus* * asymbotica*	H	East coast towns, city Q, Vic, NSW	**(4)**: Agricultural intensification: bacteria used in agricultural pest control [43]
**Origin: human **			
Dengue virus Serotypes 1–3 *	H	Q (north coast and hinterland)	**(5):** Increased residential & urban development increases artificial habitat for vector (including in dry hinterland towns with irrigated gardens) [44]
** Origin: Wildlife**			
Ross River Virus *	H D W	Expanded range Tas; urbanising Q, NSW, WA	**(5):** Creation of wetlands, reclamation or incorporation of coastal wetlands into residential developments, dryland salinity. Disease is becoming urban and involving urban adapted wildlife hosts [39,45,46,47]
Barmah Forest Virus *	H	NE to all states except Tas, SA.	**(5):** Believed similar to RRV including developments of artificial wetlands [44]
Murray Valley Encephalitis virus *	H	Endemic N WA, epidemic SE and SW	**(5):** Completion of the Argyle Dam in 1971, in Kimberleys, WA provided the opportunity for year round persistence and breeding of mosquitoes and water bird hosts, also increase in human population [48]
Flinders Island Spotted Fever *Rickettsia honei* *	H	Tas, SA, Q (Torres Straits Islands)	**(5)**: Increased human-bush exposure through residential development [49,50]
Scrub typhus, *Orientia tsuttsugamushi* new strains *	H	Northern NT, WA	**(1):** Increased recreation access to remote (rainforest) locations [51,52]
Hendra virus (flying fox to horse to human)	H D	Q (coastal)	**(4)**: Horse farms in traditionally fruit bat habitat areas. Changed virus ecology following urbanisation of fruit bats as natural habitat lost and degraded [53,54,55]
Australian Bat Lyssavirus (microbat to human)	H W	Q (coastal)	**(5):** Changed virus ecology following urbanisation of fruit bats as natural habitat lost and degraded [56,57]
Menangle virus (flying fox to pig to human)	H D	NSW (nr Sydney)	**(4)**: intensive piggery in traditional fruit bat habitat areas. Urbanisation of fruit bats, deforestation may play some role [58]
Devil Facial Tumour Disease (Tasmanian devil)	W	Tas	**(4)**: habitat converted to agricultural land, persecution leading to loss of genetic variability [59]
Amphibian Chytrid Fungus (frogs)	W	All states Tas (roads)	**(5):** Associated with dirt road construction/maintenance in Tas spreading infected amphibians, soil, and/or water [60]
**Origin: Domestic animal**			
Hydatids *E.granulosus* (dog to kangaroo to human)	H W	WA Perth	**(4);** Residential and water catchment area confluence creating new predator-prey cycles [61]
H7 Highly Pathogenic Avian Influenza (poultry)	D	Vic, NSW, Qld	**(4):** Intensification of poultry farms [62]
Newcastle Disease virulent strains (poultry)	D	NSW, Qld	**(4):** Intensification of poultry farms. Contact with wild (water) birds suspected [63]
** Locations:** Queensland (Q); New South Wales (NSW); Victoria (Vic); South Australia (SA); Tasmania (Tas); Northern Territory (NT); Western Australia (WA). ** Hosts:** Human (H), Domestic (D), Wildlife (W). **Vector-borne disease** (*) ** Land use and cover change** categorised as follows (after Lesslie *et al.*, 2010): RESIDUAL—conservation of natural state MODIFIED—minimal pastoralism, non-plantation forestry TRANSFORMED—intensive grazing and tree removal REPLACED—Agriculture, horticulture, plantations, intensive livestock production REMOVED—Development and infrastructure			

Indigenous land-use including “fire stick farming” predated European settlement in 1788 but was rapidly replaced over large areas of the country by that of the colonisers [64]. Early penal colonies and free settlements were dispersed and isolated for the first decades. Settlers then began to expand across large areas of Victoria and New South Wales (NSW) and to a lesser extent in the other states (then colonies), primarily to produce wool for export to industrial Britain. Land reforms starting from 1861 encouraged closer settlement and agriculture and reduced the size of some earlier vast land claims. Native vegetation clearing on land suitable for agriculture was actively encouraged and largely achieved in Victoria by 1890 and NSW by 1920 [27]. Rising population and prosperity saw a growth in urban development. The first commercial poultry and pig farms were established in the 1950s and 1970s respectively. Sheep production remained the dominant enterprise by land area until the 1970s [29,65]. Widespread car ownership from the 1950s opened the opportunity for sprawling outer suburbs. Peri-urban rural residential blocks followed.

Before European arrival, infectious disease with epidemic potential was largely absent from the sparse, nomadic Indigenous Australian populations [66]. Indigenous groups who came into contact with Europeans were devastated by old world diseases: smallpox, measles, tuberculosis, influenza. There appears to have been little cross-protection against venereal syphilis from pre-existing non-venereal syphilis or “yaws” [67]. Domestic animals (except the dingo and possibly the domestic cat) and their diseases were also introduced at colonisation and thereafter. Many serious livestock diseases including contagious bovine pleuropneumonia and foot and mouth disease were recognised controlled, even eradicated, before the 20th century [68,69]. The growing human population became host to the breadth of contemporary infections including diseases of childhood, close settlement and poor sanitation. Many skilled people recorded and addressed these diseases [70,71,72,73].

In the 19th century, other than malaria and scrub typhus, no infectious diseases of Indigenous people or fauna appear to have passed to Europeans or their animals, although many venoms, toxins and plants poisons were novel and infamous. “Native Pox” and “Colonial Fever” were revealed subsequently to be venereal syphilis and typhoid [70]. “Mossman Fever” and “Coastal Fever”, recognised in northern Queensland since settlement in 1877, were subsequently shown to be at least partially the result of endemic scrub typhus [74]. Malaria thwarted early attempts to settle the Northern Territory [72]. Pathogens from introduced (placental) mammals did pass to marsupials (e.g., [75,76,77]. However, the early impact of infectious disease on wildlife is unknown.

Diseases with native wildlife sources become apparent from the early 20th century. Epidemics of severe, fatal, encephalitis known as Australian X disease were first seen in 1918 at multiple locations in eastern Australia. This was later identified as Murray Valley Encephalitis Virus [48]. Ross River Virus, primarily hosted by kangaroos, is now believed to be the cause of two “unusual epidemics” of fever and arthralgia in rural NSW in the 1920s, and similar cases thereafter [78]. Rickettsial diseases Murine typhus and Queensland Tick Typhus also emerged in the early 20th century as wheat production expanded (as did mouse plagues) in the south, and large numbers of soldiers trained for World War II in the Queensland scrub [79]. Leptospirosis was first recognised in 1933 from cane cutters in north Queensland [80]. Sugar had been grown since colonisation, but by the 1930s the industry had expanded, modernised and was now exporting sugar. Buruli (Bairnsdale) Ulcer was identified in settled areas of coastal Victoria by 1948 [81]. Further zoonotic arboviruses were described from this time [82].

Reasons for the spontaneous disappearance of Dengue virus in NSW and southern Queensland around World War II include the replacement of household water storage with water piped from municipal water catchments, and the removal of stepping stones of re-watering stops for steam trains as railways converted to diesel locomotives [83]. Coordinated mosquito control began in Australia in 1959 (in Queensland) although some swamps had been drained and filled around settlements specifically for mosquito control by the turn of the 20th century. Physical modification of water flow in wetlands did not occur until the 1980s [84].

### 3.2. Discussion

Overall, LUCC was associated with 20 of the 90 EIDs reported for humans and animals in Australia since 1973. This LUCC association was more evident for EIDs of domestic animals (30%) and humans (24%) than for wildlife (11%). This is similar to a global study where almost a quarter of human EIDs were found to result from changes in land-use (18%) and agricultural intensification (13%), noting that other food production practices accounted for 13% of associations [3].

The 90 EIDs reported include many diseases of low population risk. However, of the 20 LUCC-associated EIDs, most (n = 12) have a high population risk and require serious consideration. The majority (n = 17) of the 20 LUCC-associated EIDs involved LUCC that REPLACED or REMOVED original biotic elements through agriculture, plantations, livestock or through urban development. 

The retrieval of 267 articles and 48 grey records bearing on EIDs in Australia since 1973 is high compared to literature from other countries in the region [6,85]. We believe that our large list of human EIDs reflects the greater support and resources to report on EIDs in common with other developed countries rather than a higher rate of infectious disease emergence [6]. Domestic and wild animal diseases are also well reported for Australia but are far fewer in number than that of humans. The reason for this is at least partly due to reduced effort and resources. A similar disparity between human and animal infectious diseases has been reported elsewhere [86].

The tropical north and the eastern states are the most represented among the 20 LUCC-associated EIDs, Queensland in particular. The former may reflect the higher pathogen diversity associated with low latitudes [87]. A number of arboviruses are endemic in northern Australia and have spread following ecological disturbance. The building of the vast Argyle Dam in the wet-dry tropics of the Kimberley, north Western Australia in the 1970s established permanent water and the opportunity for year round breeding of vectors and water bird hosts. This enhanced the reservoir of Murray Valley Encephalitis virus which was successfully transported south with its avian hosts with suitable weather patterns to cause a major outbreak in south eastern Australia in 1974, and intermittently since then [48].

The east coast of Australia is home to the majority of the Australian population and the majority of intensive agriculture historically and currently [29,88]. Furthermore, large areas of Queensland were mechanically cleared in the period 1960–2000 in response to lucrative export markets for agricultural products and increased demand for residential developments [40]. Indeed Queensland has been the state with the fastest growing population as well as the highest rates of land clearing in recent decades [89].

The greater frequency of LUCC-(zoonotic) EID associations in Queensland can be understood with reference to this information. Accordingly, loss of feeding grounds for flying foxes in cleared central and coastal Queensland have been proposed as a mechanism for urbanisation and changes in flying fox and virus ecology resulting in spillover of Hendra virus to horses and humans [90,91] and potentially also Australian Bat Lyssavirus [56]. The rise in autochthonous urban cases of Ross River virus in Queensland have been attributed to increased (artificial wetland) habitat for vectors [92] and urbanisation of wildlife hosts (e.g., flying foxes, brushtail possum) [45]. Urbanisation of wildlife appears to facilitates the emergence of many EIDs in this region [93]. Adaptation of wildlife to agricultural landscape change may have been important in the emergence of some earlier zoonotic diseases.

Indeed, large kangaroo species responded dramatically and positively to pastoral land change and increased water availability in early rural Australia [94]. These are major hosts of Ross River virus and were abundant and widespread by the time the first cases were described in rural New South Wales in the 1920s [78]. The breeding biology of kangaroos enables rapid activation of suspended embryos and a new, naive generation on the ground (as well as vector mosquitoes) shortly after significant rainfall events [95]. Hosts and vectors benefited from the proliferation of irregular open ground surfaces that could hold water and the hand-made and relatively shallow dams associated with agricultural and pastoral expansion.

Some diseases have emerged from ecotones, *i.e.*, edge effects such as forest clearings whereby previously separated species come into contact and may exchange pathogens. Infection with novel strains of scrub typhus in newly opened reserves in remote northern Australia [51,52], with Flinders Island Spotted Fever following residential expansion into natural environments [49], and historic emergence of scrub typhus and Queensland tick typhus [74,79] are examples of emergence at ecotones. However, the majority of EIDs described here are the result of anthropogenic landscape change facilitating changes in a native host or organism’s success, not necessarily in the same location (e.g., improved breeding opportunity for vectors; success in the urban forest for wildlife hosts displaced from native habitat; facilitation of pathogens of environmental origin; large kangaroos expanding in number and range in the pastorally transformed rangelands).

Interestingly, we have found few reports of multidirectional LUC-EID relationships. LUC is responsible for the post-war retraction of Dengue fever from NSW [83] and elimination of domestic and infrastructure water remains an important control strategy. Removal of wetlands for malaria control was progressive thinking when Dr. Edward Koch led the draining of swamps in Cairns in far north Queensland in the late 1800s. This was not followed with coordinated mosquito control until the late 1950s. This relied most heavily on personal protection, therapeutics and spraying of waterbodies near population centres, and “fogging” the centres themselves [84]. Malaria, originally endemic in northern Australia was eradicated from Australia in 1981.

Whether cessation of Indigenous “fire stick farming” had any effect on zoonotic infectious disease ecology is unknown. In many areas loss of the cumulative effect of these frequent, small, resource-management fires caused dramatic revegetation of what early Europeans had described as open parkland [64]. Increased undergrowth may support a greater number of ticks and present a similar scenario for tick-borne infectious disease reemergence such is now described for Lyme Disease in the USA. However we find no evidence of early, indigenous infectious disease emergence following this pattern in our historic review.

The historical investigation outlines disease emergence during the 185 years of European occupation of Australia before the era of EIDs in searchable literature. It demonstrates the significant volume of new infectious diseases brought to the continent in the 18th and 19th centuries, and the apparently delayed emergence of indigenous zoonotic and vector-borne diseases until the 20th century. Possible confounders of the historical relationship between indigenous zoonotic and environmental EIDs and the dramatic LUCC that preceded it (Figure 3) include improved sanitation, safer food and technical capability: these diseases may have formerly been of lesser concern or overlooked. However the ready colonial naming of endemic, and believed-to-be-endemic illnesses and the diligent reporting apparent by authorities is unlikely to have omitted such a number of significant, subsequently identified diseases.

Intervals of time with increased rates of infectious disease emergence have been described as chronotones [11]. We hypothesise that both early and late 20th century represent chronotones, and that these are associated with intense periods of LUCC. The extensive LUCC in S.E. Australia preceding the early 20th century emergence of zoonotic and environmental pathogens was concentrated over a few decades (Figure 3) [27]. Rapid LUCC in Queensland precedes the later chronotone. More specifically, our analysis of LUCC-EID relationships finds that the final stages of LUCC transition, the ecological state shift, dominate.

State shifts are resistant to return to their prior state. LUC-EID associations may reflect this as a threshold effect or sustained EID risk. We have discussed above the persistent effects of such LUC on wildlife hosts and vectors. Removing these new patterns of host-pathogen-vector associations would result in further ecological change; return to original ecosystem states is unlikely. The coastal wetland development associated with Buruli ulcer reemergence [37,38]; the establishment of commercial native forests from the 1990s—including the important host of EID-causing *Cryptococcus gattii*, the River Red Gum (now one of the most extensively planted forestry trees in the world) [42] and the expansion of intensive livestock and agricultural industries, are all new systems from which new risks (continue) to emerge. The emergence of virulent Newcastle disease and highly pathogenic avian influenza in poultry, zoonotic bat—origin Menangle virus disease in pigs and horticultural pest biological control agent and EID *Photorabdus asymbiotica,* are consequences of the shift to intensive food production [43,58,62,63].

These diseases can also be discussed as outcomes of social-ecological systems [17]. However by limiting our discussion to ecological drivers—here the quantifiable, LUCC aspect of LUC, we may better understand the pervasive nature of landscape change on EIDs and focus research hypotheses and subsequent interventions at an ecosystem level [14]. This entails abundant complexity of its own. As both the analysis and the examples given illustrate, LUC associated EID risk may act at a population or individual level, and/or at different spatial scales.

One unavoidable constraint for our study is the basis of evidence for LUCC-EID associations. Evidence was based on ecological associations that are biologically plausible and temporarily sequenced with the LUCC preceding the EID. These are two key criteria for inferring a causal association [96]. It was not possible to quantify the strength of the association or degree of attribution between LUCC and the emergence of related EIDs. Indeed, such calculations are usually not appropriate for characterising relationships that are expressions of complex and distally acting ecological processes [12,97]. Such challenges call for novel approaches to conceptualising and studying these associations. Here we address this challenge by setting our study within an historical perspective of infectious disease emergence and LUCC, and within one continent with a common (although not homogenous) biogeographic, political, and social-economic system. In this way we appreciate the epidemiological ‘life course’ as well as ‘life stage’ of LUCC associated infectious disease risk in Australia [15].

The majority of LUCC-EID associations identified in this study, act at a regional scale of variable dimensions. This is less problematical for proximal or near-proximal associations. For example, the (introduced) amphibian chytridmycosis pathogen has been shown to be spread into pristine areas by dirt road construction and maintenance in Tasmanian Wilderness World Heritage Area [60]. Hydatids, an important rural zoonoses (sheep-dog cycles) is increasingly recognised in Perth, W.A. as a periurban disease maintained in converged sylvatic(kangaroo)-domestic(dog) cycles amongst leafy suburbs and integrated reserves [61]. However historic and contemporary LUCC at variable scales has also accumulated as contemporary disease risk. The autogenous, infectious facial tumours that have decimated the distribution and abundance of Tasmanian Devils have emerged in a species with significantly reduced genetic variability and long the target of persecution and displacement [59].

Our use of LUCC categories from a standardised Australian system of vegetation cover change [32] has the advantage of reflecting vegetation communities at abstract scale although it is essentially geographic. This system was created using an assumed pre-1750 vegetation condition benchmark based on knowledge of the effects of land-use and land management practices on vegetation. As such it may be a source of misclassification bias. In our conceptual use of this system this potential source of bias is unlikely to be important. More specific spatial information than found in the literature reviewed in this study is required to realise the potential of this data. The relevant scale of individual LUCC-EID relationships requires specific research.

Another unavoidable constraint of this study was the need to confine the systematic review and analysis to the period from 1973. This was a consequence of the birth of the “emerging infection” search term and concept at that time. The work of many great veterinarians and public health doctors is consequently less visible than it should be, but can be glimpsed through the historical perspective included in this study.

This study, with its specific focus on the influence of categorised LUC (as LUCC) on EID, is not easily compared with other reports. There are few reports explicitly incorporating this topic and the criteria vary substantially. For example, in one report, LUC is regarded as separate to agricultural intensification and food production [3]. The American Institute of Medicine grouped LUC with economic development but distinct from changing ecosystems [1]. Other authors have summarised mechanisms of LUC to include changes to the physical environment; movement of populations, pathogens, and trade; agriculture; and urbanisation [2]. In addition, the pattern of LUC here described for Australia and the response of its unique flora and fauna cannot be replicated.

Global concern with EIDs is focused on the period since 1973. Land transformation has been extensive and accelerated over this period globally [5] but it is not uniform. In many countries this period corresponds with a period of dramatic changes in patterns and intensity of land-use, whilst in others there has been improving environmental management and ecological restoration. LUCC-EID associations are unlikely to be independent of the background rate and pattern of LUCC prior to the 1970s, or of multi-scale and multi-directional effects. Our historical review of LUC and EID in continental Australia provides an example of a prior interval of zoonotic and environmental pathogen emergence following dramatic ecological change. Certainly, recent emergence of diseases due to LUCC is part of a longer trend starting at least by the early 20th century in Australia. An aggregation of other country and continental case studies would improve our understanding of these relationships as a global phenomenon.

Further spatial and disease transmission research on the specific LUCC-EID associations is required to inform land-use planning for these diseases. Regulating the proximity of intensive livestock production units to human centres, wildlife areas and to each other has been nominated as a useful strategy to limit emergence and spread of livestock and zoonotic EIDs [98]. However, even strong, proximal associations between coastal marsh development, artificial wetlands and risk of Ross River virus currently do little to dissuade developers in Australia [92,46]. Proposals to move agriculture to higher rainfall zones in northern Australia (in response to changing climate patterns), and to restore native vegetation connectivity across rural landscapes (to address declining biodiversity) are examples of new forces by which land use, and the ecology of vectors, hosts and pathogens in Australia may change in the future. As a significant proportion of EIDs are currently associated with LUCC it would be short sighted not to monitor such changes closely.

## 4. Conclusions

We have highlighted the experience of a single continent with its unique biogeography, and compressed history of modern development. The systematic assessment of LUCC-EID relationships has demonstrated associations for 22% of human and animal EIDs in Australia. EIDs with LUCC associations most frequently occur with the REPLACEMENT and REMOVAL of natural vegetation (*i.e.*, agriculture, plantations and intensive livestock production; settlements and infrastructure). These two categories are the most advanced stages of LUCC and represent an ecological state shift. Temporal associations between LUCC and disease emergence can be demonstrated early in the 20th century in Australia and in the era of EIDs (1973–). These periods of increased rates of infectious disease emergence follow major periods of native vegetation clearing. Direct comparison with other regions is difficult because of complex and location specific biotic and abiotic factors and varying prior rates and extents of ecological change. Comparisons would also require the use of a similar definition of LUC. However the unique example of Australia provides insight into other less easily studied regions and supports a growing concern that global land-use and ecological change is an important driver of EIDs.

## Supplementary Files

Supplementary File 1 Supplementary Information (PDF, 172 KB)
