# Supplementary material for: Land-Use Change and Emerging Infectious Disease on an Island Continent
Source: Int J Environ Res Public Health. 2013 Jun 28;10(7):2699–719. doi: 10.3390/ijerph10072699 (PMC3734451; doi:10.3390/ijerph10072699)
Supplement: Supplementary File 1 — Supplementary Information (PDF, 172 KB) [file ijerph-10-02699-s001.pdf]

Land-Use Change and Emerging Infectious Disease on an Island Continent

Table 1. Emerging infectious diseases of humans and animals of Australia (n = 90) derived from a systematic review of literature 1970–December 2009.

**Emerging infections of humans**
**Human diseases (or pathogens)of environmental (or invertebrate) origin**
Acinetobacter baumannii [1]
Bairnsdale-, Daintree- , Buruli ulcer *Mycobacteria ulcerans* * [2,3]
Cryptococcus gattii [4]
Fungal diseases in the immuno-compromised Candida, Aspergillosis, *Rhodotorula* spp., Mucor, Paecilomyces, Zygomycetes, Fusarium, *Scedosporium apiospermum* [5,6]
Legionaires disease *Legionella* spp. [7]
Melioidosis *Burkholderia pseudomallei* * [8,9]
Photorhabdus asymbiotica [10]
Trachipleistophora hominis (Human Microsporidia) [11]
Vibrio vulnificus ** [12]
**Human to Human diseases (or pathogens)**
Influenza A H1N1 "Swine Flu" [13]
Enterovirus 71 Hand Foot and Mouth Disease [14]
HIV AIDS [15]
Metapneumovirus ** [16]
Rhinovirus A2 ** [17]
Norovirus (strain GGII.4) [18]
HCoV-NL63 human coronavirus ** [19]
Heliobacter pylori Peptic Ulcer Disease ** [20]
Mycobacterium haemophilum [21]
Rotavirus G9 serotype G4P[6] and G9P[8] strains [22]
Cyclospora cayatenensis ("Travellers Diarrohea") [23]
Pertussis/Whooping cough** [20]
Rubella ** [24]
Entamoeba histolytica [25]
Hepatitis C [26]
Antibiotic resistant bacteria:
a. *Staphlococcus aureus*
CA-MRSA Community acquired multi-drug resistant *S. aureus* [27,28]
ORSA Oxacillin resistant *S. aureus* infections [29]
MDRSA multidrug resistant *S. aureus* inc Vancomyacin [28]
b. Penicillin-resistant pneumococci [30]
c. Vancomycin-resistant enterococci (VRE) [30]
d. Extended-spectrum beta-lactamase (ESBL) resistant *Klebsiella pneumoniae* [31]

Table 1. *Cont.*

**Human Vector-Borne Diseases (or pathogens)**
Dengue serotypes 1–3 * [32]
Ross River Virus, urbanising* [33–36]
Barmah Forest Virus [32,37]
Japanese encephalitis [32,37]
Murray Valley Encephalitis * [32,38]
Kunjin Virus [32]
Flinders Island Spotted fever *Rickettsia honei* [39,40]
Australian spotted fever *Rickettsia marmionii* [41]
Scrub typhus * *Orientia tsutsugamushi*, new strains [42,43]
Note also the potentially zoonotic arboviruses:
Edge Hill, Trubanaman, GanGan, Alfuy, Sindbis, Kokobera, Stratford viruses [32]
**Human food- and water- borne diseases (or pathogens)**
Cryptosporidium parvum [20]
Campylobacterosis [44]
Non-typhoid Salmonellosis [44]
Giardiasis (*G. duodenalis*) [45]
Shigatoxigenic/Verotoxigenic *E. coli* (inc 0157H7& 0111) [44]
Hepatitis E ** [46]
Yersinia enterocolitica * [47]
**Other zoonotic diseases (or pathogens) of domestic animal origin**
Ancylostoma caninum [48]
Cross species acquired methicillen resistant *Staphlococcus aureus* [49]
Hydatids (*Echinococcus granulosus*) * (urbanising.canine) [50]
**Zoonotic diseases (or pathogens) of wildlife origin**
Hendra Virus [51]
Australian Bat Lyssavirus [52,53]
Menangle Virus [54,55]
Brucella suis (feral pig) [56]
Angiostrongiliasis (rat lungworm) [57]
**EMERGING INFECTIONS OF ANIMALS**
**Wildlife Diseases (or pathogen) of environmental origin**
Cryptococcosis *Cryptococcus gattii* (Koalas) [4]

Table 1. *Cont.*

**Livestock and domestic animal diseases of single species**
Equine Influenza (horse) [58]
Tritrichomonas cf. foetus. (cat) [59]
Canine infectious cyclic thrombocytopenia *Anaplasma platys* [60]
**Wildlife single species diseases (or pathogens)**
Tasmanian Devil Facial Tumour Disease [61,62]
Rabbit calicivirus/ haemorrhagic disease [63]
Koala retrovirus [64,65]
**Livestock and domestic animal diseases (or pathogens)of multiple species (10)**
Angiostrongiliasis (multiple spp.) [66,67]
Highly Pathogenic Avian influenza H7N3,4,7 [68]
Hendra virus (horses) [51,67,69]
Menangle virus (pigs) [54]
Porcine Myocarditis (pigs) [70]
Newcastle Disease (highly virulent strains) [71]
Cross species acquired methicillen resistant Staphlococcus aureus [49]
Neospora caninum ** [72]
Canine parvovirus CPV 2, 2a, 2b [73]
Note also reported equine infection Ross River Virus [74]
porcine infection with Japanese Encephalitis [75]
**Wildlife diseases (or pathogens) involving multiple species**
Angiostrongiliasis rat lungworm (multiple spp. affected) [66]
Avian Papiloma Virus (parrots) [76]
Australian Bat Lyssavirus (bats) [52,53]
Airsacculitis *Sternostoma tracheacolum (*wild Gouldian Finches) [77]
Bohle virus (frog) [78]
Chytridimycosis (frogs) [79,80]
Inclusion Body Disease of Boids (snakes) [81]
Mucormycosis (platypus) [82,83]
Ophidian paramyxoviruses (snakes) [84]
Proventricular dilatation disease (parrots) [76,85]
Pacheco’s Disease (parrots) [76]
Wamena virus (snake) [86]
**Vector-borne diseases of wildlife**
Cutaneous leishmanianiasis (kangaroo) [87]
Chorioretinitis (kangaroo) [88,89]
Note also: brush tail possum clinical pathology associated with Ross River Virus [33]

Notes: * re-emerging,** re-identified

References

1. Peleg, A.Y.; Seifert, H.; Paterson, D.L. *Acinetobacter baumannii*: Emergence of a successful pathogen. *Clin. Microbiol. Rev.* **2008**, *21*, 538–582.
2. Johnson, P.; Azuolas, J.; Lavender, C.; Wishart, E.; Stinear, T.; Hayman, J.; Brown, L.; Jenkin, G.; Fyfe, J. *Mycobacterium ulcerans* in mosquitoes captured during outbreak of Buruli ulcer, Southeastern Australia. *Emerg. Infect. Dis.* **2007**, *13*, 1653–1660.
3. Veitch, M.; Johnson, P.; Flood, P.; Leslie, D.; Street, A.; Hayman, J. A large localized outbreak of *Mycobacterium ulcerans* infection on a temperate southern Australian island. *J. Epidemiol. Infect.* **1997**, *119*, 313–318.
4. Sorrell, T.C. *Cryptococcus* neoformans variety gattii. *Med. Mycol.* **2001**, *39*, 155–168.
5. Delhaes, L. Molecular typing of Australian Scedosporium isolates showing genetic variability and numerous *S. aurantiacum. Emerg. Infect. Dis.* **2008**, *14*, 282–290.
6. Richardson, M.; Lass-Flörl, C. Changing epidemiology of systemic fungal infections. *Clin. Microbiol. Infect.* **2008**, *14*, 5–24.
7. Greig, J.; Carnie, J.; Tallis, G.; Zwolak, B.; Hart, W.G.; Guest, C.S.; Ryan, N.J.; Leydon, J.A.; Tan, A.G.; Gordon, I.R. An outbreak of Legionnaires' disease at the Melbourne Aquarium, April 2000: Investigation and case-control studies. *Med. J. Aust.* **2004**, *180*, 566–572.
8. Currie, B.J.; Fisher, D.A.; Howard, D.M.; Burrow, J.N.C.; Selvanayagam, S.; Snelling, P.L.; Anstey, N.M.; Mayo, M.J. The epidemiology of melioidosis in Australia and Papua New Guinea. *Acta Tropica* **2000**, *74*, 121–127.
9. Kaestli, M.; Mayo, M.; Harrington, G.; Ward, L.; Watt, F.; Hill, J.V.; Cheng, A.C.; Currie, B.J. Landscape changes influence the occurrence of the melioidosis bacterium *Burkholderia pseudomallei* in soil in Northern Australia. *PLoS Negl. Trop. Dis.* **2009**, *3*, doi:10.1371/journal.pntd.0000364.
10. Gerrard, J.; Joyce, S.A.; Clarke, D.J.; ffrench-Constant, R.H.; Nimmo, G.R.; Looke, D.F.M.; al., Nematode symbiont for *Photorhabdus asymbiotica*. *Emerg. Infect. Dis.* **2006**, *6*, 229–237.
11. Mathis, A.; Weber, R.; Deplazes, P. Zoonotic potential of the microsporidia. *Clin. Microbiol. Rev.* **2005**, *18*, 423–445.
12. Ralph, A.; Currie, B. *Vibrio vulnificus* and *V. parahaemolyticus* necrotising fasciitis in fishermen visiting an estuarine tropical northern Australian location. *J. Infect.* **2007**, *54*, e111–e114.
13. Bishop, J.F.; Murnane, M.P.; Owen, R. Australia's winter with the 2009 pandemic influenza A (H1N1) virus. *N. Engl. J. Med.* **2009**, *361*, 2591–2594.
14. Daley, A.J.; Dwyer, D.E. Emerging viral infections in Australia. *J. Paediatr. Child Health* **2002**, *38*, 1–3.
15. Howse, G. Managing emerging infectious diseases: Is a federal system an impediment to effective laws? *Aust. New Zealand Health Policy* **2004**, *1*, doi: 2010.1186/1743-8462-2001-2007.
16. Principi, N.; Bosis, S.; Esposito, S. Human metapneumovirus in paediatric patients. *Clin. Microbiol. Infect.* **2006**, *12*, 301–308.
17. Mackay, I.M.; Lambert, S.B.; McErlean, P.K.; Faux, C.E.; Arden, K.E.; Nissen, M.D.; Sloots, T.P. Prior evidence of putative novel Rhinovirus species, Australia. *Emerg. Infect. Dis.* **2008**, *14*, 1823–1824.

18. Verhoef, L.; Depoorterej, E.; Boxman, I.; Duizer, E.; van Duynhoven, Y.; Harris, J.; Johnsen, C.; Kroneman, A.; Le Guyader, S.; Lim, W.; *et al.* Emergence of new norovirus variants on spring cruise ships and prediction of winter epidemics. *Emerg. Infect. Dis.* **2008**, *14,* 238–243.
19. Arden, K.E.; Nissen, M.D.; Sloots, T.P.; Mackay, I.M. New human coronavirus, HCoV-NL63, associated with severe lower respiratory tract disease in Australia. *J. Med. Virol.* **2005**, *75*, 455-462.
20. Longbottom, H. Emerging infectious diseases. *Commun. Dis. Intell.* **1997**, *21*, 89–93.
21. Saubolle, M.A.; Kiehn, T.E.; White, M.H.; Rudinsky, M.F.; Armstrong, D. *Mycobacterium haemophilum*: Microbiology and expanding clinical and geographic spectra of disease in humans. *Clin. Microbiol. Rev.* **1996**, *9*, 435–437.
22. Kirkwood, C.; Bogdanovic-Sakran, N.; Barnes, G.; Bishop, R. Rotavirus serotype G9P[8] and acute gastroenteritis outbreak in children, Northern Australia. *Emerg. Infect. Dis.* **2004**, *10*, 1593–1600.
23. James, S.L. Emerging parasitic infections. *FEMS Immunol. Med. Microbiol.* **1997**, *18*, 313–317.
24. Cheffins, T.; Chan, A.; Keane, R.J.; Haan, E.A.; Hall, R. The impact of rubella immunisation on the incidence of rubella, congenital rubella syndrome and rubella-related terminations of pregnancy in South Australia. *Br. J. Obstetrics Gynaecol.* **1998**, *105*, 998–1004.
25. Stark, D.; Hal, S.J.V.; Matthews, G.; Harkness, J.; Marriott, D. Invasive amebiasis in men who have sex with men, Australia. *Emerg. Infect. Dis.* **2008**, *14*, 1141–1143.
26. Dodd, R.Y. Emerging infections and global blood safety. *Adv. Transfus. Saf.* **2007**, *127*, 237–248.
27. Collignon, P.; Nimmo, G.R.; Gottlieb, T.; Gosbell, I.B. *Staphylococcus aureus* bacteremia, Australia. *Emerg. Infect. Dis.* **2005**, *11*, 554–561.
28. Coombs, G.W.; Pearson, J.C.; O'Brien, F.G.; Murray, R.J.; Grubb, W.B.; Christiansen, K.J. Methicillin-resistant *Staphylococcus aureus* clones, Western Australia. *Emerg. Infect. Dis.* **2006**, *12*, 241–247.
29. Gosbell, I.B.; Mercer, J.L.; Neville, S.A.; Chant, K.G.; Munro, R. Community-acquired, non-multiresistant oxacillin-resistant *Staphylococcus aureus* (NORSA) in South Western Sydney. *Pathology* **2001**, *33*, 206–210.
30. Beaman, M.H. Emerging infections in Australia. *Ann. Acad. Med. Singapore* **1997**, *26*, 609–615.
31. Paterson, D.L.; Ko, W.C.; Von Gottberg, A.; Mohapatra, S.; Casellas, J.M.; Goossens, H.; Mulazimoglu, L.; Trenholme, G.; Klugman, K.P.; Bonomo, R.A.; *et al.* International prospective study *Klebsiella pneumoniae* bacteremia: Implications of extended-spectrum beta-lactamase production in nosocomial infections. *Ann. Inter. Med.* **2004**, *140*, 26–32.
32. Russell, R.; Dwyer, D. Arboviruses associated with human disease in Australia. *Microb. Infect.* **2000**, *2*, 1693–1704.
33. Boyd, A.; Hall, R.A.; Gemmell, R.T.; Kay, B. Experimental infection of Australian brushtail possums, *Trichosurus vulpecula* (Phalangeridae:Marsupialia), with Ross River and Barmah Forest viruses by use of a natural mosquito vector system. *Am. J. Trop. Med. Hyg.* **2001**, *65*, 777–782.
34. Brokenshire, T.; Symmonds, D.; Reynolds, R.; Doggett, S.; Geary, M.; Russell, R. A cluster of locally-acquired Ross River virus infection in outer Western Sydney. *New South Wales Public Health Bull.* **2000**, *11*, 132–134.
35. Carver, S.; Spafford, H.; Storey, A.; Weinstein, P. Dryland salinity and the ecology of Ross River Virus: The ecological underpinnings of the potential for transmission. *Vect. Borne Zoon. Dis.* **2009**, *9*, 611–622.

36. Lindsay, M. An outbreak of Ross River Disease in southwestern Australia. *Emerg. Infect. Dis.* **1996**, *2*, 117–120.
37. Mackenzie, J.S.; Chua, K.B.; Daniels, P.W.; Eaton, B.T.; Field, H.E.; Hall, R.A.; Halpin, K.; Johansen, C.A.; Kirkland, P.D.; Lam, S.K.; *et al.* Emerging viral diseases of Southeast Asia and the Western Pacific. *Emerg. Infect. Dis.* **2001**, *7*, 497–504.
38. Marshall, I.D. Murray Valley Encephalitis. In *Encyclopedia of Arthropod-Transmitted Infections of Man and Domesticated Animals*; Service, M.W., Ashford, R W, Eds. CABI: Oxford, UK, 2001; pp. 346–255.
39. Raoult, D.; Roux, V. Rickettsioses as paradigms of new or emerging infectious diseases. *Clin. Microbiol. Rev.* **1997**, *10*, 694–719.
40. Unsworth, N.B.; Stenos, J.; Graves, S.R.; Faa, A.G.; Cox, G.E.; Dyer, J.R.; Boutlis, C.S.; Lane, A.M.; Shaw, M.D.; Robson, J.; *et al.* Flinders Island spotted fever rickettsioses caused by "marmionii" strain of *Rickettsia honei*, Eastern Australia. *Emerg. Infect. Dis.* **2007**, *13*, 566–573.
41. Dyer, J.R.; Einsiedel, L.; Ferguson, P.E.; Lee, A.S.; Unsworth, N.B.; Graves, S.R.; Gordon, D.L. A new focus of *Rickettsia honei* spotted fever in South Australia. *Med. J. Aust.* **2005**, *182*, 231–235.
42. Odorico, D.; Graves, S.; Currie, B.; Catmull, J.; Nack, Z.; Ellis, S.; Wang, L.; Miller, D. New Orientia tsutsugamushi Strain from Scrub Typhus in Australia. *Emerg. Infect. Dis.* **1998**, *4*, 641–644.
43. Ralph, A.; Raines, M.; Whelan, P.; Currie, B.J. Scrub typhus in the Northern Territory: Exceeding the boundaries of Litchfield National Park. *Commun. Dis. Intell. Q. Rep.* **2004**, *28*, 267–269.
44. Hall, G.V.; D'Souza, R.M.; Kirk, M.D. Foodborne disease in the new millennium: Out of the frying pan and into the fire? *Med. J. Aust.* **2002**, *177*, 614–618.
45. Thompson, R.C.A. Giardiasis as a re-emerging infectious disease and its zoonotic potential. *Int. J. Parasitol.* **2000**, *30*, 1259–1267.
46. Dalton, H.R.; Bendall, R.; Ijaz, S.; Banks, M. Hepatitis E: An emerging infection in developed countries. *Lancet Infect. Dis.* **2008**, *8*, 698–709.
47. Tauxe, R.V. Emerging foodborne diseases: An evolving public health challenge. *Emerg. Infect. Dis.* **1997**, *3*, 425–434.
48. Macpherson, C.N.L. Human behaviour and the epidemiology of parasitic zoonoses. *Int. J. Parasitol.* **2005**, *35*, 1319–1331.
49. Barton , M.D. Antimicrobial resistance in veterinary medicine—Issues and controversies from an Australian perspective *Microbiol. Aust.* **2007**, *2007*, 179–181.
50. Jenkins, D.J.; Romig, T.; Thompson, R.C.A. Emergence/re-emergence of *Echinococcus* spp.—A global update. *Int. J. Parasitol.* **2005**, *35*, 1205–1219.
51. Field, H.; Young, P.; Yob, J.M.; Mills, J.; Hall, L.; Mackenzie, J. The natural history of Hendra and Nipah viruses. *Microb. Infect.* **2001**, *3*, 307–314.
52. Hooper, P.T.; Lunt, R.A.; Gould, A.R.; Samaratunga, H.; Hyatt, A.D.; Gleeson, L.J.; Rodwell, B.J.; Rupprecht, C.E.; Smith, J.S.; Murray, P.K. A new lyssavirus—The first endemic rabies-related virus recognized in Australia. *Bulletin de l'Institut Pasteur* **1997**, *95*, 209–218.
53. McCall, B.; Epstein, J.H.; Neill, A.S.; Heel, K.; Field, H.; Barrett, J.; Smith, G.A.; Selvey, L.A.; Rodwell, B.; Lunt, R. Potential human exposure to Australian bat lyssavirus, Queensland, 1996–1999. *Emerg. Infect. Dis.* **2000**, *6*, 259–264.

54. Philbey, A.W.; Kirkland, P.D.; Ross, A.D.; Field, H.E.; Srivastava, M.; Davis, R.J.; Love, R. Infection with Menangle virus in flying foxes (*Pteropus* spp.) in Australia. *Aust. Veter. J.* **2008**, *86*, 449–454.
55. Westbury, H.A. Classical Swine Fever and Emerging Diseases in Southeast Asia. In *Emerging Viruses of Animals in Australia and Southeast Asia*, Blacksell, S.D., Ed.; Australian Centre for International Agricultural Research (ACIAR): Canberra, Australia, 2000; pp. 15–20.
56. Robson, J.; Harrison, M.W.; Wood, R.N.; Tilse, M.H.; McKay, A.B.; Brodribb, T.R. Brucellosis: Re-emergence and changing epidemiology in Queensland. *Med. J. Aust.* **1993**, *159*, 153–158.
57. Prociv, P.; Spratt, D.M.; Carlisle, M.S. Neuro-angiostrongylus: Unresolved issues. *Int. J. Parasitol.* **2000**, *30*, 1295–1303.
58. Cowled, B.; Ward, M.P.; Hamilton, S.; Garner, G. The equine influenza epidemic in Australia: Spatial and temporal descriptive analyses of a large propagating epidemic. *Prev. Veter. Med.* **2009**, *92*, 60–70.
59. Bissett, S.A.; Gowan, R.A.; O'Brien, C.R.; Stone, M.R.; Gookin, J.L. Feline diarrhoea associated with Tritrichomonas *cf.* foetus and Giardia co-infection in an Australian cattery. *Aust. Veter. J.* **2008**, *86*, 440–443.
60. Brown, G.; Martin, A.; Roberts, T.; Aitken, R. Detection of *Ehrlichia platys* in dogs in Australia. *Aust. Veter. J.* **2001**, *79*, 554–558.
61. Jones, M.E.; Jarman, P.J.; Lees, C.M.; Hesterman, H.; Hamede, R.K.; Mooney, N.J.; Mann, D.; Pukk, C.E.; Bergfeld, J.; McCallum, H. Conservation management of tasmanian devils in the context of an emerging, extinction-threatening disease: Devil facial tumor disease. *Ecohealth* **2007**, *4*, 326–337.
62. Obendorf, D.; McGlashan, N. Research priorities in the Tasmanian devil facial tumour debate. *Eur. J. Oncol.* **2008**, *13*, 229–238.
63. White, P.J.; Norman, R.A.; Hudson, P.J. Epidemiological consequences of a pathogen having both virulent and avirulent modes of transmission: The case of rabbit haemorrhagic disease virus. *Epidemiol. Infect.* **2002**, *129*, 665–677.
64. Tarlington, R.; Meers, J.; Young, P. Retroviral invasion of the Koala genome. *Nature* **2006**, *442*, 79–81.
65. Stoye, J.P. Koala retrovirus: A genome invasion in real time. *Genome Biol.* **2006**, *7*, doi:10.1186/gb-2006-7-11-241.
66. Spratt, D. Australian ecosystems, capricious food chains and parasitic consequences for people. *Int. J. Parasitol.* **2005**, *35*, 717–724.
67. Plowright, R.K.; Sokolow, S.H.; Gorman, M.E.; Daszak, P.; Foley, J.E. Causal inference in disease ecology: Investigating ecological drivers of disease emergence. *Front. Ecol. Environ.* **2008**, *6*, 420–429.
68. Westbury, H.A. History of highly pathogenic avian influenza in Australia and the H7N3 outbreak 1995. In Proceedings of the 4th International Symposium on Avian Influenza, Athens, GA, USA, 1998; pp. 22–30.
69. Field, H. Ecology of Hendra Virus and Australian Bat Lyssavirus. Doctoral Thesis, University of Queensland, Brisbane, Australia, 2005.

70. Finlaison, D.; King, K.; Frost, M.; Kirkland, P. Field and laboratory evidence that Bungowannah virus, a recently recognised pestivirus, is the causative agent of the porcine myocarditis syndrome (PMC). *Veter. Microbiol.* **2009**, *136*, 259–265.
71. Westbury, H. Newcastle disease virus: An evolving pathogen? *Avian Pathol.* **2001**, *30*, 5–11.
72. Reichel, M. *Neospora caninum* infections in Australia and New Zealand. *Austr. Veter. J.* **2000**, *78*, 258–261.
73. Kapil, S.; Yeary, T.; Johnson, B. Diagnostic investigation of emerging viruses of companion animals. *Veter. Clin. North Am.* **2008**, *38*, 755–774.
74. Azuolas, J.K.; Wishart, E.; Bibby, S.; Ainsworth, C. Isolation of Ross River virus from mosquitoes and from horses with signs of musculo-skeletal disease. *Aust. Veter. J.* **2003**, *81*, 344–347.
75. Hanna, J.; Ritchie, S.; Phillips, D.; Shield, J.; Bailey, M.; Mackenzie, J.; Poidinger, M.; McCall, B.; Mills, P. An outbreak of Japanese encephalitis in the Torres Strait, Australia, 1995. *Med. J. Aust.* **1996**, *165*, 256–260.
76. PROMED Avian diseases, imported—Australia. Available online: http://www.promedmail.org/ (accessed on 1 June 2012).
77. Tidemann, S.C.; McOrist, S.; Woinarski, J.C.; Freeland, W.J. Parasitism of wild Gouldian finches (*Erythrura gouldiae*) by the air-sac mite *Sternostoma tracheacolum*. *J. Wildlife Dis.* **1992**, *28*, 80–84.
78. Speare, R.; Smith, J. An irido virus like agent isolated from the ornate burrowing frog *Lymnodynastes ornatus* in Northern Australia. *Dis. Aquat. Org.* **1992**, *14*, 51–57.
79. Berger, L.; Speare, R.; Daszak, P.; Earl Greene, D.; Cunningham, A.A.; Gogging, L.; Slocombe, R.; Ragani, M.A.; Hyatt, A.D.; McDonald, K.R.; *et al.* Chytridiomycosis causes amphibian mortality associated with population declines in the rainforests of Australia and Central America. *Proc. Natl. Acad. Sci.* **1998**, *95*, 9031–9036.
80. Pauza, M.; Driessen, M. *Distribution and Potential Spread of Amphibian Chytrid Fungus Batrachochytrium Dendrobatidis in the Tasmanian World Heritage Area*; Department of Primary Industries and Water, Biodiversity Conservation Branch: Tasmania, 2008.
81. Carlisle-Nowak, M.S.; Sullivan, N.; Carrigan, N.; Knight, C.; Ryan, C.; Jacobson, E.R. Inclusion body disease in two captive australian pythons (*Morelia spilota* variegata and *Morelia spilota* spilota). *Aust. Veter. J.* **1998**, *76*, 98–100.
82. Munday, B.; Peel, B. Severe ulcerative dermatitis in platypus (*Ornithorhynchus anatinus*). *J. Wildlife Dis.* **1983**, *19*, 263–365.
83. Obendorf, D.L.; Peel, B.F.; Munday, B.L. *Mucor amphibiorum* infection in platypus (*Ornithorhynchus anatinus*) from Tasmania. *J. Wildlife Dis.* **1993**, *29*, 485–487.
84. *Epidemiological Review of Wildlife Diseases*; OIE: Geneva, Switzerland, 2004.
85. Doneley, R.J.T.; Miller, R.I.; Fanning, T.E. Proventricular dilatation disease: An emerging exotic disease of parrots in Australia. *Aust. Veter. J.* **2007**, *85*, 119–123.
86. Hyatt, A.D.; Williamson, M.; Coupar, B.E.H.; Middleton, D.; Hengstberger, S.G.; Gould, A.R.; Selleck, P.; Wise, T.G.; Kattenbelt, J.; Cunningham, A.A.; Lee, J. First identification of a ranavirus from green pythons (*Chondropython viridis*). *J. Wildlife Dis.* **2002**, *38*, 239–252.
87. Rose, K.; Curtis, J.; Baldwin, T.; Mathis, A.; Kumar, B.; Sakthianandeswaren, A.; Spurck, T.; Low Choy, J.; Handman, E. *Cutaneous leishmaniasis* in red kangaroos: Isolation and characterisation of the causative organisms. *Int. J. Parasitol.* **2004**, *34*, 655–664.

88. Hooper, P. Kangaroo blindness and some other new viral diseases in Australia. *Aust. Veter. J.* **1999**, *77*, 514–515.
89. Reddacliff, L.; Kirkland, P.; Philbey, A.; Davis, R.; Vogelness, L.; Hulst, F.; Blyde, D.; Deykin, A.; Smith, J.; Hooper, P. Experimental reproduction of viral chorioretinitis in kangaroos. *Aust. Veter. J.* **1999**, *77*, 522–528.

Appendix 1. Historical sources used in the study

Infectious Disease Sources:

1. Australian Society of the History of Medicine. History, Heritage and Health In Proceedings of the Fourth Biennial Conference. Covacevich, J., Pearn, J., Case, D., Chapple, I., Phillips, G., Eds.; Norfolk Island, 2–9 July 1995; ASHM: Brisbane, Australia, 1995.
2. Bolderwood, R.S.W. *Silvers and Co.'s Australian Grazier's Guide I (1879) II (1889)*; University of New England: Armidale, Australia, 1994.
3. Cumpston, J.H.L.; Lewis, M.J. *Health and Disease in Australia: A History*; AGPS: Canberra, Australia, 1989.
4 Derrick, E. Q Fever—A new fever identity: Clinical features, diagnosis and laboratory investigation. *Med. J. Aust.* **1937**, *2*, 281–299.
5. Derrick, E. The challenge of North Queensland Fevers. *Aust. Ann. Med.* **1957**, *6*, 173–188.
6. Dowling, P.J. A Great Deal of Sickness—Introduced Diseases among the Aboriginal People of Colonial Southeast Australia 1788–1900. Ph.D. Thesis, Australian National University, Canberra, Australia, 1997.
7. Emanuel, M.L.I.; Mackerras, I.M.; Smith, D.J.W. The epidemiology of leptospirosis in North Queensland: I. General survey of animal hosts. *J. Hyg.* **1964**, *62*, 451–484.
8. *History of Microbiology in Australia*; Fenner, F., Ed.; Australian Society for Microbiology, Brolga Press: Canberra, Australia, 1990.
9. Gandevia, B. The pattern of Australian medical history. *Proc. Royal Soc. Melbourne* **1957**, *50*, 591–598.
10. Graves, S.; Stenos, J. Rickettsioses in Australia. *Ann. New York Acad. Sci.* **2009**, *1166*, 151–155.
11. *Australian Colonial Medicine*; Hagger, J., Ed.; Rigby: Adelaide, Australia, 1979.
12. Hayman, J. Beyond the Barcoo—Probable human tropical cyanobacterial poisoning in outback Australia. *Med. J. Aust.* **1992**, *157*, 794–796.
13. Heaslip, W. Tsutsugamushi fever in north Queensland, Australia. *Med. J. Aust.* **1941**, *1*, 380–392.
14. Izzard, L. Rickettsiales and Rickettsial Diseases in Australia. Ph.D. Thesis, Murdoch University: Perth, Australia, 2010.
15. *The Peoples Health: Public Health in Australia 1788–1950*; Lewis, M.J., Ed.; Praeger: Santa Barbara, CA, USA, 2003.
16. *Paradise of Quacks. An alternative history of Medicine in Australia*; Martyr, P., Ed.; Macleay Press: Sydney, Australia, 2002.
17. Mathew, R. Interim notes on an outbreak of coastal fever at Tully, North Queensland. *Health* **1934**, *12*, 54–57.
18. *Pioneer Medicine in Australia*; Pearn, J., Ed.; Amphion Press: Brisbane, Australia, 1988.

19. *Fevers and Frontiers*; Pearn, J., Cobcroft, M., Eds.; Amphion Press: Brisbane, Australia, 1990.
20. *A Companion of the History of Medicine In Australia 1788–1939*; Proust, A., Ed.; Proust: Canberra, Australia, 2003.
21. Thearle, M. People, places and pestilence. Vignettes of Queensland's Medical Past; University Department of Child Health, Mater Childrens Hospital: South Brisbane, Australia, 1986.
22. Turner, A.J. Endemic disease control and regulation in Australia 1901–2010. *Aust. Veter. J.* **2011**, *89*, 413–421.
23. Turner, A.J. Disease control during the colonial period in Australia. *Aust. Veter. J.* **2011**, *89*, 239–242.
24. Webb, S. Infectious Disease; In *Palaeopathology of Aboriginal Australians: Health and Disease across a Hunter-Gatherer Continent*; Cambridge University Press: Cambridge, UK, 2009; Chapter 6, pp.125–161.

Population, Land and Vegetation Sources:

1. Australian Bureau of Statistics. A Picture of the Nation; Australian Government Printing Service: Canberra, Australia, 2006. Available online: http://www.abs.gov.au/AUSSTATS/abs@.nsf/DetailsPage/2070.02006?OpenDocument (accessed on 1 June 2012).
2. Australian Bureau of Statistics. Australian Historical Population Statistics; Australian Government Printing Service: Canberra, Australia, 2012. Available online: http://www.abs.gov.au/ausstats/abs@.nsf/mf/3105.0.65.001 (accessed on 1 June 2012).
3. Australian Chicken Meat Federation Inc. History of the Industry. Available online: http://www.chicken.org.au/index.php (accessed on 1 August 2012).
4. Bradshaw, C. Little left to lose: Deforestation and forest degradation in Australia since European colonization. *Plant Ecol.* **2012**, *5*, 109–120.
5. Bureau of Resource Science. Land Use Change, Productivity and Diversification. Final Report of Theme 5.1 to the National Land & Water Resources Audit; Australian Government Printing Service: Canberra, Australia, 2001.
6. Bureau of Resource Science. *Land Use in Australia at a Glance*; Australian Government Printing Service: Canberra, Australia, 2006.
7. Department of the Environment and Water Resources. *Australia's Native Vegetation: A Summary of Australia's Major Vegetation Groups, 2007.* Australian Government: Canberra, Australia, 2007.
8. Durack, M. *Kings in Grass Castles*; Constance & Co.: London, UK, 1959.
9. Gammage, B. *The Biggest Estate on Earth—How Aborigines Made Australia*; Allen and Unwin: Sydney, Australia, 2011.
10. Henzell, T. *Australian Agriculture: Its History and Challenges*; CSIRO: Melbourne, Australia, 2007.
11. Kingsford, R.; Watson, J.; Lundquist, C.; Venter, O.; Hughes, L.; Johnston, E.; Atherton, J.; Gawell, M.; Keith, D.; Mackey, B.; *et al.* Major conservation policy issues for biodiversity in oceania. *Conserv. Biol.* **2009**, *23*, 834–840.
12. Newsome, A.E. An ecological comparison of the two arid-zone kangaroos of Australia, and their anomalous prosperity since the introduction of ruminant stock to their environment. *Quar. Rev. Biol.* **1975**, *50*, 389–424.

13. Noble, I.; Barson, M.; Dumsday, R.; Friedel, M.; Hacker, R.; McKenzie, N.; Smith, G.; Young, M.; Maliel, M.; Zammit, G. Land Resources in State of the Environment Report 1996; Environment Australia, Australian Government Printing Service: Canberra, Australia, 1996.
14. Pickard, J. Shepherding in Colonial Australia. *Rural His.* **2008**, *19*, 55–80.
15. Rolls, E. *A Million Wild Acres*; Nelson: Melbourne, Australia, 1981.
16. Rolls, E. *They All Ran Wild: The Animals and Plants that Plague Australia*; Angus and Robertson: Sydney, Australia, 1984.

© 2013 by the authors; licensee MDPI, Basel, Switzerland. This article is an open access article distributed under the terms and conditions of the Creative Commons Attribution license (http://creativecommons.org/licenses/by/3.0/).
